# 3-(Adamantan-1-yl)-4-ethyl-1-[(4-phenyl­piperazin-1-yl)meth­yl]-1*H*-1,2,4-triazole-5(4*H*)-thione

**DOI:** 10.1107/S160053681202990X

**Published:** 2012-07-07

**Authors:** Ali A. El-Emam, Ebtehal S. Al-Abdullah, Hanaa M. Al-Tuwaijri, Mohammed Said-Abdelbaky, Santiago García-Granda

**Affiliations:** a College of Pharmacy, King Saud University, PO Box 2457, Riyadh 11451, Saudi Arabia; b Departamento de Química Física y Analítica, Facultad de Química, Universidad de Oviedo – CINN, C/ Julián Clavería, 8, 33006 Oviedo, Asturias, Spain

## Abstract

The title compound, C_25_H_35_N_5_S, has an approximately C-shaped conformation. The dihedral angle between the triazole and phenyl planes is 79.5 (2)°. The crystal structure consists of infinite chains parallel to the *b* axis, constructed by C—H⋯S hydrogen bonds between translation-related mol­ecules. Adjacent chains are linked *via* weak C—H⋯C inter­actions between the adamantyl and phenyl groups.

## Related literature

For the biological activity of adamantane derivatives and adamantyl-1,2,4-triazoles, see: Vernier *et al.* (1969 ▶); Al-Deeb *et al.* (2006 ▶); Al-Omar *et al.* (2010 ▶); El-Emam & Ibrahim (1991 ▶); El-Emam *et al.* (2004 ▶); Kadi *et al.* (2007 ▶, 2010 ▶). For related adamantyl-1,2,4-triazole structures, see: Al-Tamimi *et al.* (2010 ▶); Al-Abdullah *et al.* (2012 ▶); El-Emam *et al.* (2012 ▶); Lahsasni *et al.* (2012 ▶).
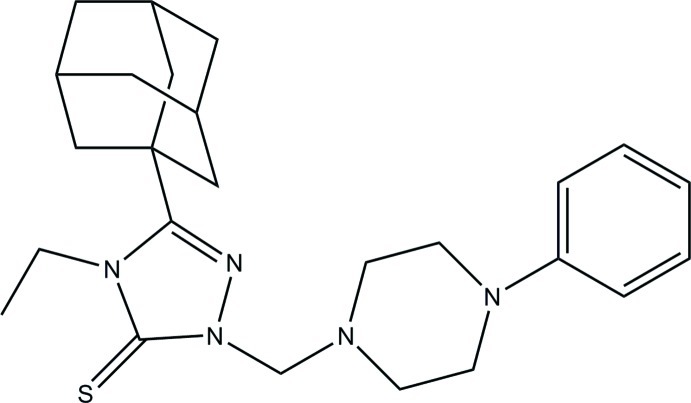


## Experimental

### 

#### Crystal data

C_25_H_35_N_5_S
*M* *_r_* = 437.65
Orthorhombic, 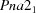

*a* = 27.382 (4) Å
*b* = 6.5083 (7) Å
*c* = 13.369 (2) Å
*V* = 2382.4 (5) Å^3^
*Z* = 4
Cu *K*α radiation
μ = 1.36 mm^−1^
*T* = 293 K
0.16 × 0.06 × 0.02 mm

#### Data collection

Oxford Diffraction Xcalibur Gemini R diffractometer
Absorption correction: multi-scan (*CrysAlis PRO*; Oxford Diffraction, 2010 ▶) *T*_min_ = 0.919, *T*_max_ = 1.000
5844 measured reflections
3016 independent reflections
1828 reflections with *I* > 2σ(*I*)
*R*_int_ = 0.086

#### Refinement

*R*[*F* ^2^ > 2σ(*F* ^2^)] = 0.057
*wR*(*F* ^2^) = 0.128
*S* = 1.00
3016 reflections
282 parameters
1 restraint
H-atom parameters constrained
Δρ_max_ = 0.17 e Å^−3^
Δρ_min_ = −0.18 e Å^−3^
Absolute structure: Flack (1983 ▶), 632 Friedel pairs
Flack parameter: 0.00 (4)

### 

Data collection: *CrysAlis CCD* (Oxford Diffraction, 2010 ▶); cell refinement: *CrysAlis RED* (Oxford Diffraction, 2010 ▶); data reduction: *CrysAlis RED*; program(s) used to solve structure: *SIR92* (Altomare *et al.*, 1994 ▶); program(s) used to refine structure: *SHELXL97* (Sheldrick, 2008 ▶); molecular graphics: *ORTEP-3 for Windows* (Farrugia, 1997 ▶) and *Mercury* (Macrae *et al.*, 2008 ▶); software used to prepare material for publication: *WinGX* (Farrugia, 1999 ▶) and *publCIF* (Westrip, 2010 ▶).

## Supplementary Material

Crystal structure: contains datablock(s) global, I. DOI: 10.1107/S160053681202990X/fy2061sup1.cif

Structure factors: contains datablock(s) I. DOI: 10.1107/S160053681202990X/fy2061Isup2.hkl

Supplementary material file. DOI: 10.1107/S160053681202990X/fy2061Isup3.cml

Additional supplementary materials:  crystallographic information; 3D view; checkCIF report

## Figures and Tables

**Table 1:** Hydrogen-bond geometry (Å, °)

*D*—H⋯*A*	*D*—H	H⋯*A*	*D*⋯*A*	*D*—H⋯*A*
C15—H15*A*⋯S1^i^	0.97	2.90	3.836 (5)	162
C5—H5*A*⋯C20^ii^	0.97	2.80	3.750 (6)	167

Symmetry codes: (i) 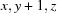; (ii) 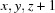.

**Fig. 1.**
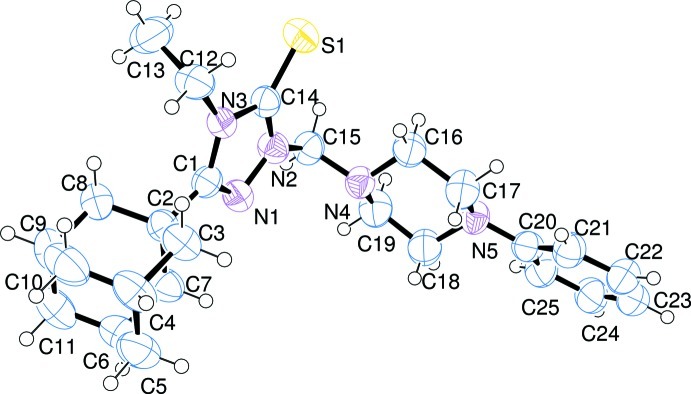
ORTEP-style plot of title compound with labeling. Ellipsoids are given at the 50% probability level.

## supplementary crystallographic information

### Comment

Adamantane derivatives were early recognized for their diverse biological activities including antiviral activity against the influenza (Vernier *et al.*, 1969) and HIV viruses (El-Emam *et al.*, 2004). In addition, adamantane derivatives were reported to exhibit marked antibacterial (Kadi *et al.*, 2007, 2010) and anti-inflammatory (El-Emam & Ibrahim, 1991) activities. In continuation to our interest in the chemical and pharmacological properties of adamantane derivatives, we synthesized the title compound (I) as a potential bioactive agent. The structure consists of infinite chains parallel to the *b* axis, constructed by translations of a single molecule. The molecules in the the same chain are connected through C—H···S interactions with a H···S distance of 2.90 Å. Moreover, chains are linked *via* the weak C5—H5B···C20 interaction with a bond distance of 2.80 Å. The plane of the 1,2,4-triazole ring includes the *S*,*C*(ethyl group), *C* (adamantyl group) and C15 substituent atoms with deviations from the L.S. plane (in Å) of 0.0582, -0.1062, 0.0568 and -0.0964, respectively. The phenyl ring plane includes atom N5 with a deviation of 0.0668 Å. The angle between these two planes is 79.5 (2)°.

### Experimental

A mixture of 527 mg (2 mmol) of 3-(1-adamantyl)-4-ethyl-4*H*-1,2,4-triazole-5-thiol (El-Emam & Ibrahim, 1991), 1-phenylpiperazine (325 mg, 2 mmol) and 37% formaldehyde solution (1 ml), in ethanol (8 ml), was heated under reflux for 15 min when a clear solution was obtained. Stirring was continued for 12 h at room temperature and the mixture was allowed to stand overnight. Cold water (5 ml) was slowly added and the mixture was stirred for 20 min. The precipitated crude product was filtered, washed with water, dried, and crystallized from ethanol to yield 770 mg (88%) of the title compound (C_25_H_35_N_5_S) as colorless needle crystals. M.P.: 139–141°C. Single crystals suitable for X-ray analysis were obtained by slow evaporation of CHCl_3_:EtOH solution (1:1; 5 ml) at room temperature. ^1^H NMR (CDCl_3_, 500.13 MHz): δ 1.13 (t, 3H, CH_2_CH_3_, J = 7.0 Hz), 1.67–1.73 (m, 6H, Adamantane-H), 1.96 (s, 6H, Adamantane-H), 2.03 (s, 3H, Adamantane-H), 2.88 (s, 4H, Piperazine-H), 3.09 (s, 4H, Piperazine-H), 4.17 (q, 2H, CH_2_CH_3_, J = 7.0 Hz), 5.08 (s, 2H, CH_2_), 6.46–6.83 (m, 3H, Ar—H), 7.15–7.17 (m, 2H, Ar—H). ^13^C NMR (CDCl_3_, 125.76 MHz): δ 13.81 (CH_2_CH_3_), 27.95, 35.24, 36.31, 39.90 (Adamantane-C), 43.43 (CH_2_CH_3_), 49.40, 50.37 (Piperazine-C), 68.80 (CH_2_), 116.32, 119.99, 129.12, 151.27 (Ar—C), 156.10 (Triazole C-5), 168.75 (C=S).

### Refinement

Carbon-bound H-atoms were placed in calculated positions (C—H 0.95 to 0.98 Å) and were included in the refinement in the riding model approximation, with U_iso_(H) set to 1.2 or 1.5 (for methyl groups) U_eq_(C).

### Crystal data

**Table:**

C_25_H_35_N_5_S	*F*(000) = 944
*M**_r_* = 437.65	*D*_x_ = 1.220 Mg m^−^^3^
Orthorhombic, *P**n**a*2_1_	Cu *K*α radiation, λ = 1.54184 Å
Hall symbol: P 2c -2n	Cell parameters from 728 reflections
*a* = 27.382 (4) Å	θ = 3.7–70.5°
*b* = 6.5083 (7) Å	µ = 1.36 mm^−^^1^
*c* = 13.369 (2) Å	*T* = 293 K
*V* = 2382.4 (5) Å^3^	Prism, colourless
*Z* = 4	0.16 × 0.06 × 0.02 mm

### Data collection

**Table:**

Oxford Diffraction Xcalibur Gemini R diffractometer	3016 independent reflections
Radiation source: Enhance (Cu) X-ray Source	1828 reflections with *I* > 2σ(*I*)
Graphite monochromator	*R*_int_ = 0.086
Detector resolution: 10.2673 pixels mm^-1^	θ_max_ = 70.7°, θ_min_ = 4.6°
ω scans	*h* = −27→32
Absorption correction: multi-scan (*CrysAlis PRO*; Oxford Diffraction, 2010)	*k* = −7→7
*T*_min_ = 0.919, *T*_max_ = 1.000	*l* = −9→16
5844 measured reflections	

### Refinement

**Table:**

Refinement on *F*^2^	Secondary atom site location: difference Fourier map
Least-squares matrix: full	Hydrogen site location: inferred from neighbouring sites
*R*[*F*^2^ > 2σ(*F*^2^)] = 0.057	H-atom parameters constrained
*wR*(*F*^2^) = 0.128	*w* = 1/[σ^2^(*F*_o_^2^) + (0.0292*P*)^2^] where *P* = (*F*_o_^2^ + 2*F*_c_^2^)/3
*S* = 1.00	(Δ/σ)_max_ = 0.001
3016 reflections	Δρ_max_ = 0.17 e Å^−^^3^
282 parameters	Δρ_min_ = −0.18 e Å^−^^3^
1 restraint	Absolute structure: Flack (1983), 632 Friedel pairs
Primary atom site location: structure-invariant direct methods	Flack parameter: 0.00 (4)

### Special details

**Table:**

Geometry. All e.s.d.'s (except the e.s.d. in the dihedral angle between two l.s. planes) are estimated using the full covariance matrix. The cell e.s.d.'s are taken into account individually in the estimation of e.s.d.'s in distances, angles and torsion angles; correlations between e.s.d.'s in cell parameters are only used when they are defined by crystal symmetry. An approximate (isotropic) treatment of cell e.s.d.'s is used for estimating e.s.d.'s involving l.s. planes.
Refinement. Refinement of *F*^2^ against ALL reflections. The weighted *R*-factor *wR* and goodness of fit *S* are based on *F*^2^, conventional *R*-factors *R* are based on *F*, with *F* set to zero for negative *F*^2^. The threshold expression of *F*^2^ > σ(*F*^2^) is used only for calculating *R*-factors(gt) *etc*. and is not relevant to the choice of reflections for refinement. *R*-factors based on *F*^2^ are statistically about twice as large as those based on *F*, and *R*- factors based on ALL data will be even larger.

### Fractional atomic coordinates and isotropic or equivalent isotropic displacement parameters (Å^2^)

**Table:**

	*x*	*y*	*z*	*U*_iso_*/*U*_eq_	
S1	0.47530 (6)	0.0990 (2)	0.88657 (13)	0.0661 (4)	
N1	0.40642 (17)	0.4993 (7)	1.0508 (3)	0.0559 (11)	
N2	0.42569 (16)	0.4251 (7)	0.9622 (3)	0.0533 (11)	
N3	0.44288 (16)	0.2016 (7)	1.0746 (3)	0.0495 (10)	
N4	0.37594 (16)	0.5467 (7)	0.8271 (4)	0.0536 (11)	
N5	0.30519 (17)	0.5670 (7)	0.6693 (3)	0.0540 (11)	
C1	0.41610 (19)	0.3589 (8)	1.1182 (4)	0.0486 (12)	
C2	0.39947 (19)	0.3789 (8)	1.2243 (4)	0.0498 (12)	
C3	0.3646 (2)	0.1977 (9)	1.2515 (5)	0.0634 (15)	
H3A	0.3370	0.1958	1.2060	0.076*	
H3B	0.3819	0.0681	1.2452	0.076*	
C4	0.3465 (3)	0.2256 (11)	1.3600 (5)	0.0765 (19)	
H4	0.3253	0.1100	1.3776	0.092*	
C5	0.3177 (2)	0.4225 (11)	1.3669 (6)	0.0805 (19)	
H5A	0.3045	0.4378	1.4339	0.097*	
H5B	0.2906	0.4186	1.3202	0.097*	
C6	0.3508 (3)	0.6039 (11)	1.3427 (5)	0.0764 (19)	
H6	0.3323	0.7322	1.3478	0.092*	
C7	0.3692 (2)	0.5759 (9)	1.2349 (5)	0.0671 (16)	
H7A	0.3416	0.5701	1.1897	0.081*	
H7B	0.3891	0.6932	1.2162	0.081*	
C8	0.4414 (2)	0.3859 (11)	1.2987 (4)	0.0672 (16)	
H8A	0.4627	0.4999	1.2824	0.081*	
H8B	0.4602	0.2601	1.2937	0.081*	
C9	0.4223 (3)	0.4110 (12)	1.4070 (5)	0.082 (2)	
H9	0.4499	0.4153	1.4537	0.099*	
C10	0.3897 (3)	0.2300 (12)	1.4314 (5)	0.092 (2)	
H10A	0.3781	0.2413	1.4997	0.110*	
H10B	0.4081	0.1033	1.4254	0.110*	
C11	0.3934 (3)	0.6097 (11)	1.4138 (5)	0.089 (2)	
H11A	0.3815	0.6282	1.4816	0.107*	
H11B	0.4143	0.7250	1.3975	0.107*	
C12	0.4659 (2)	0.0198 (9)	1.1198 (5)	0.0650 (17)	
H12A	0.4599	−0.0992	1.0779	0.078*	
H12B	0.4514	−0.0060	1.1848	0.078*	
C13	0.5207 (2)	0.0501 (14)	1.1319 (6)	0.096 (3)	
H13A	0.5352	−0.0745	1.1563	0.143*	
H13B	0.5267	0.1593	1.1786	0.143*	
H13C	0.5348	0.0847	1.0683	0.143*	
C14	0.4485 (2)	0.2439 (8)	0.9739 (4)	0.0487 (12)	
C15	0.42363 (18)	0.5519 (8)	0.8724 (4)	0.0561 (14)	
H15A	0.4318	0.6926	0.8895	0.067*	
H15B	0.4477	0.5031	0.8246	0.067*	
C16	0.3704 (2)	0.3810 (9)	0.7556 (5)	0.0640 (15)	
H16A	0.3784	0.2514	0.7875	0.077*	
H16B	0.3929	0.4013	0.7004	0.077*	
C17	0.3181 (2)	0.3728 (9)	0.7154 (5)	0.0670 (17)	
H17A	0.3153	0.2632	0.6666	0.080*	
H17B	0.2957	0.3436	0.7699	0.080*	
C18	0.3126 (2)	0.7352 (11)	0.7401 (5)	0.077 (2)	
H18A	0.2907	0.7184	0.7964	0.092*	
H18B	0.3049	0.8646	0.7078	0.092*	
C19	0.3642 (2)	0.7407 (10)	0.7770 (5)	0.0707 (17)	
H19A	0.3863	0.7619	0.7211	0.085*	
H19B	0.3683	0.8540	0.8234	0.085*	
C20	0.2619 (2)	0.5739 (9)	0.6120 (4)	0.0587 (14)	
C21	0.2322 (2)	0.4026 (11)	0.5996 (4)	0.0650 (16)	
H21	0.2393	0.2810	0.6331	0.078*	
C22	0.1913 (2)	0.4140 (12)	0.5361 (5)	0.0732 (18)	
H22	0.1719	0.2983	0.5260	0.088*	
C23	0.1798 (2)	0.5956 (12)	0.4890 (5)	0.0775 (19)	
H23	0.1523	0.6035	0.4482	0.093*	
C24	0.2089 (3)	0.7651 (12)	0.5023 (5)	0.0766 (19)	
H24	0.2006	0.8880	0.4711	0.092*	
C25	0.2496 (2)	0.7563 (10)	0.5605 (4)	0.0688 (16)	
H25	0.2695	0.8714	0.5665	0.083*	

### Atomic displacement parameters (Å^2^)

**Table:**

	*U*^11^	*U*^22^	*U*^33^	*U*^12^	*U*^13^	*U*^23^
S1	0.0731 (8)	0.0694 (8)	0.0558 (8)	0.0032 (8)	0.0078 (9)	−0.0078 (9)
N1	0.061 (3)	0.059 (3)	0.048 (3)	0.003 (2)	0.000 (2)	−0.004 (2)
N2	0.059 (2)	0.056 (3)	0.045 (2)	0.000 (2)	0.003 (2)	0.003 (2)
N3	0.053 (2)	0.050 (2)	0.046 (2)	0.002 (2)	−0.003 (2)	−0.005 (2)
N4	0.056 (2)	0.053 (3)	0.052 (2)	0.001 (2)	−0.006 (2)	0.001 (2)
N5	0.058 (3)	0.048 (2)	0.056 (3)	−0.001 (2)	−0.010 (2)	0.004 (2)
C1	0.048 (3)	0.046 (3)	0.051 (3)	−0.001 (2)	−0.005 (3)	0.005 (2)
C2	0.051 (3)	0.047 (3)	0.052 (3)	−0.001 (3)	−0.002 (3)	−0.003 (2)
C3	0.073 (4)	0.059 (3)	0.058 (4)	−0.006 (3)	0.010 (3)	−0.007 (3)
C4	0.102 (5)	0.066 (4)	0.062 (4)	−0.014 (4)	0.024 (4)	−0.007 (3)
C5	0.078 (4)	0.087 (5)	0.077 (5)	−0.008 (4)	0.023 (4)	−0.015 (4)
C6	0.089 (4)	0.070 (4)	0.070 (4)	0.012 (4)	0.016 (4)	−0.011 (3)
C7	0.085 (4)	0.054 (3)	0.063 (4)	0.011 (3)	0.008 (3)	−0.004 (3)
C8	0.066 (3)	0.082 (4)	0.055 (4)	−0.000 (3)	−0.005 (3)	−0.011 (3)
C9	0.090 (4)	0.106 (5)	0.051 (4)	0.007 (5)	−0.012 (4)	−0.012 (4)
C10	0.133 (7)	0.087 (5)	0.056 (4)	0.025 (5)	0.017 (5)	0.003 (4)
C11	0.117 (6)	0.078 (4)	0.072 (5)	−0.007 (5)	0.012 (4)	−0.030 (3)
C12	0.082 (4)	0.057 (3)	0.056 (3)	0.018 (3)	0.007 (4)	0.010 (3)
C13	0.072 (4)	0.143 (7)	0.072 (4)	0.038 (5)	0.004 (4)	0.019 (5)
C14	0.048 (3)	0.055 (3)	0.043 (3)	−0.006 (3)	0.001 (3)	0.000 (2)
C15	0.058 (3)	0.063 (3)	0.048 (3)	−0.002 (3)	−0.000 (3)	0.010 (3)
C16	0.071 (3)	0.049 (3)	0.072 (4)	0.005 (3)	−0.010 (3)	−0.004 (3)
C17	0.077 (4)	0.051 (3)	0.073 (4)	−0.002 (3)	−0.015 (3)	0.006 (3)
C18	0.083 (4)	0.063 (4)	0.084 (5)	0.015 (4)	−0.019 (4)	−0.009 (4)
C19	0.084 (4)	0.058 (4)	0.070 (4)	0.000 (4)	−0.012 (4)	0.005 (3)
C20	0.059 (3)	0.062 (3)	0.055 (3)	0.006 (3)	0.001 (3)	−0.001 (3)
C21	0.061 (3)	0.071 (4)	0.062 (4)	−0.006 (3)	−0.002 (3)	0.003 (3)
C22	0.061 (3)	0.089 (5)	0.070 (4)	0.001 (4)	−0.006 (3)	−0.005 (4)
C23	0.069 (4)	0.099 (6)	0.064 (4)	0.020 (4)	−0.011 (3)	−0.011 (4)
C24	0.086 (5)	0.082 (5)	0.062 (4)	0.023 (4)	−0.018 (4)	−0.001 (4)
C25	0.077 (4)	0.064 (4)	0.065 (4)	0.007 (3)	−0.012 (4)	0.006 (3)

### Geometric parameters (Å, º)

**Table:**

S1—C14	1.670 (6)	C9—C11	1.520 (10)
N1—C1	1.311 (7)	C9—H9	0.9800
N1—N2	1.384 (6)	C10—H10A	0.9700
N2—C14	1.344 (7)	C10—H10B	0.9700
N2—C15	1.459 (7)	C11—H11A	0.9700
N3—C14	1.383 (7)	C11—H11B	0.9700
N3—C1	1.388 (7)	C12—C13	1.522 (9)
N3—C12	1.471 (7)	C12—H12A	0.9700
N4—C15	1.440 (7)	C12—H12B	0.9700
N4—C16	1.449 (7)	C13—H13A	0.9600
N4—C19	1.465 (7)	C13—H13B	0.9600
N5—C20	1.412 (7)	C13—H13C	0.9600
N5—C17	1.450 (8)	C15—H15A	0.9700
N5—C18	1.461 (8)	C15—H15B	0.9700
C1—C2	1.495 (8)	C16—C17	1.530 (8)
C2—C8	1.518 (8)	C16—H16A	0.9700
C2—C7	1.533 (8)	C16—H16B	0.9700
C2—C3	1.560 (8)	C17—H17A	0.9700
C3—C4	1.544 (8)	C17—H17B	0.9700
C3—H3A	0.9700	C18—C19	1.496 (9)
C3—H3B	0.9700	C18—H18A	0.9700
C4—C5	1.509 (9)	C18—H18B	0.9700
C4—C10	1.520 (10)	C19—H19A	0.9700
C4—H4	0.9800	C19—H19B	0.9700
C5—C6	1.525 (9)	C20—C21	1.390 (8)
C5—H5A	0.9700	C20—C25	1.413 (8)
C5—H5B	0.9700	C21—C22	1.408 (9)
C6—C11	1.504 (10)	C21—H21	0.9300
C6—C7	1.538 (9)	C22—C23	1.376 (10)
C6—H6	0.9800	C22—H22	0.9300
C7—H7A	0.9700	C23—C24	1.373 (10)
C7—H7B	0.9700	C23—H23	0.9300
C8—C9	1.548 (9)	C24—C25	1.361 (9)
C8—H8A	0.9700	C24—H24	0.9300
C8—H8B	0.9700	C25—H25	0.9300
C9—C10	1.514 (11)		
			
C1—N1—N2	105.6 (4)	C6—C11—C9	110.2 (6)
C14—N2—N1	112.6 (4)	C6—C11—H11A	109.6
C14—N2—C15	127.6 (5)	C9—C11—H11A	109.6
N1—N2—C15	119.5 (4)	C6—C11—H11B	109.6
C14—N3—C1	108.7 (4)	C9—C11—H11B	109.6
C14—N3—C12	120.9 (5)	H11A—C11—H11B	108.1
C1—N3—C12	130.3 (4)	N3—C12—C13	111.2 (5)
C15—N4—C16	112.9 (5)	N3—C12—H12A	109.4
C15—N4—C19	111.8 (5)	C13—C12—H12A	109.4
C16—N4—C19	108.5 (5)	N3—C12—H12B	109.4
C20—N5—C17	117.6 (5)	C13—C12—H12B	109.4
C20—N5—C18	116.4 (5)	H12A—C12—H12B	108.0
C17—N5—C18	110.1 (5)	C12—C13—H13A	109.5
N1—C1—N3	109.4 (5)	C12—C13—H13B	109.5
N1—C1—C2	122.0 (5)	H13A—C13—H13B	109.5
N3—C1—C2	128.6 (5)	C12—C13—H13C	109.5
C1—C2—C8	113.2 (4)	H13A—C13—H13C	109.5
C1—C2—C7	108.9 (5)	H13B—C13—H13C	109.5
C8—C2—C7	108.8 (5)	N2—C14—N3	103.6 (5)
C1—C2—C3	110.0 (4)	N2—C14—S1	128.2 (4)
C8—C2—C3	109.4 (5)	N3—C14—S1	128.1 (4)
C7—C2—C3	106.3 (5)	N4—C15—N2	111.6 (4)
C4—C3—C2	109.0 (5)	N4—C15—H15A	109.3
C4—C3—H3A	109.9	N2—C15—H15A	109.3
C2—C3—H3A	109.9	N4—C15—H15B	109.3
C4—C3—H3B	109.9	N2—C15—H15B	109.3
C2—C3—H3B	109.9	H15A—C15—H15B	108.0
H3A—C3—H3B	108.3	N4—C16—C17	110.8 (5)
C5—C4—C10	110.7 (6)	N4—C16—H16A	109.5
C5—C4—C3	109.0 (6)	C17—C16—H16A	109.5
C10—C4—C3	110.0 (6)	N4—C16—H16B	109.5
C5—C4—H4	109.0	C17—C16—H16B	109.5
C10—C4—H4	109.0	H16A—C16—H16B	108.1
C3—C4—H4	109.0	N5—C17—C16	110.3 (5)
C4—C5—C6	109.4 (5)	N5—C17—H17A	109.6
C4—C5—H5A	109.8	C16—C17—H17A	109.6
C6—C5—H5A	109.8	N5—C17—H17B	109.6
C4—C5—H5B	109.8	C16—C17—H17B	109.6
C6—C5—H5B	109.8	H17A—C17—H17B	108.1
H5A—C5—H5B	108.2	N5—C18—C19	111.3 (5)
C11—C6—C5	110.3 (6)	N5—C18—H18A	109.4
C11—C6—C7	110.0 (5)	C19—C18—H18A	109.4
C5—C6—C7	107.6 (6)	N5—C18—H18B	109.4
C11—C6—H6	109.6	C19—C18—H18B	109.4
C5—C6—H6	109.6	H18A—C18—H18B	108.0
C7—C6—H6	109.6	N4—C19—C18	109.7 (6)
C2—C7—C6	111.2 (5)	N4—C19—H19A	109.7
C2—C7—H7A	109.4	C18—C19—H19A	109.7
C6—C7—H7A	109.4	N4—C19—H19B	109.7
C2—C7—H7B	109.4	C18—C19—H19B	109.7
C6—C7—H7B	109.4	H19A—C19—H19B	108.2
H7A—C7—H7B	108.0	C21—C20—N5	122.0 (5)
C2—C8—C9	111.2 (5)	C21—C20—C25	118.4 (5)
C2—C8—H8A	109.4	N5—C20—C25	119.4 (6)
C9—C8—H8A	109.4	C20—C21—C22	119.7 (6)
C2—C8—H8B	109.4	C20—C21—H21	120.1
C9—C8—H8B	109.4	C22—C21—H21	120.1
H8A—C8—H8B	108.0	C23—C22—C21	120.2 (7)
C10—C9—C11	110.0 (7)	C23—C22—H22	119.9
C10—C9—C8	108.5 (6)	C21—C22—H22	119.9
C11—C9—C8	108.8 (6)	C24—C23—C22	119.9 (6)
C10—C9—H9	109.9	C24—C23—H23	120.0
C11—C9—H9	109.9	C22—C23—H23	120.0
C8—C9—H9	109.9	C25—C24—C23	121.1 (7)
C9—C10—C4	109.8 (6)	C25—C24—H24	119.5
C9—C10—H10A	109.7	C23—C24—H24	119.5
C4—C10—H10A	109.7	C24—C25—C20	120.6 (7)
C9—C10—H10B	109.7	C24—C25—H25	119.7
C4—C10—H10B	109.7	C20—C25—H25	119.7
H10A—C10—H10B	108.2		
			
C1—N1—N2—C14	−1.9 (6)	C10—C9—C11—C6	−58.7 (7)
C1—N1—N2—C15	−176.4 (5)	C8—C9—C11—C6	60.0 (8)
N2—N1—C1—N3	2.4 (6)	C14—N3—C12—C13	74.2 (7)
N2—N1—C1—C2	−177.8 (5)	C1—N3—C12—C13	−101.9 (7)
C14—N3—C1—N1	−2.2 (6)	N1—N2—C14—N3	0.5 (6)
C12—N3—C1—N1	174.2 (5)	C15—N2—C14—N3	174.4 (5)
C14—N3—C1—C2	178.0 (5)	N1—N2—C14—S1	178.2 (4)
C12—N3—C1—C2	−5.6 (9)	C15—N2—C14—S1	−7.9 (9)
N1—C1—C2—C8	−118.6 (6)	C1—N3—C14—N2	1.0 (6)
N3—C1—C2—C8	61.1 (8)	C12—N3—C14—N2	−175.9 (5)
N1—C1—C2—C7	2.5 (7)	C1—N3—C14—S1	−176.7 (4)
N3—C1—C2—C7	−177.7 (5)	C12—N3—C14—S1	6.4 (8)
N1—C1—C2—C3	118.7 (6)	C16—N4—C15—N2	−88.9 (6)
N3—C1—C2—C3	−61.5 (7)	C19—N4—C15—N2	148.5 (5)
C1—C2—C3—C4	−178.0 (5)	C14—N2—C15—N4	107.8 (6)
C8—C2—C3—C4	57.1 (7)	N1—N2—C15—N4	−78.7 (6)
C7—C2—C3—C4	−60.2 (7)	C15—N4—C16—C17	175.9 (5)
C2—C3—C4—C5	62.4 (7)	C19—N4—C16—C17	−59.7 (6)
C2—C3—C4—C10	−59.2 (7)	C20—N5—C17—C16	168.3 (5)
C10—C4—C5—C6	58.4 (7)	C18—N5—C17—C16	−55.2 (7)
C3—C4—C5—C6	−62.7 (8)	N4—C16—C17—N5	57.9 (7)
C4—C5—C6—C11	−58.6 (7)	C20—N5—C18—C19	−165.7 (5)
C4—C5—C6—C7	61.4 (8)	C17—N5—C18—C19	57.2 (7)
C1—C2—C7—C6	179.7 (5)	C15—N4—C19—C18	−174.4 (5)
C8—C2—C7—C6	−56.5 (7)	C16—N4—C19—C18	60.4 (6)
C3—C2—C7—C6	61.2 (6)	N5—C18—C19—N4	−59.8 (7)
C11—C6—C7—C2	58.1 (8)	C17—N5—C20—C21	0.2 (8)
C5—C6—C7—C2	−62.1 (7)	C18—N5—C20—C21	−133.6 (6)
C1—C2—C8—C9	178.9 (5)	C17—N5—C20—C25	−176.0 (6)
C7—C2—C8—C9	57.6 (7)	C18—N5—C20—C25	50.2 (7)
C3—C2—C8—C9	−58.2 (7)	N5—C20—C21—C22	−175.6 (5)
C2—C8—C9—C10	59.9 (8)	C25—C20—C21—C22	0.6 (8)
C2—C8—C9—C11	−59.7 (8)	C20—C21—C22—C23	−2.1 (9)
C11—C9—C10—C4	58.0 (7)	C21—C22—C23—C24	1.3 (10)
C8—C9—C10—C4	−60.8 (8)	C22—C23—C24—C25	1.0 (10)
C5—C4—C10—C9	−58.6 (7)	C23—C24—C25—C20	−2.5 (10)
C3—C4—C10—C9	61.9 (8)	C21—C20—C25—C24	1.7 (9)
C5—C6—C11—C9	58.9 (7)	N5—C20—C25—C24	178.0 (6)
C7—C6—C11—C9	−59.6 (8)		

### Hydrogen-bond geometry (Å, º)

**Table:**

*D*—H···*A*	*D*—H	H···*A*	*D*···*A*	*D*—H···*A*
C15—H15*A*···S1^i^	0.97	2.90	3.836 (5)	162
C5—H5*A*···C20^ii^	0.97	2.80	3.750 (6)	167

Symmetry codes: (i) *x*, *y*+1, *z*; (ii) *x*, *y*, *z*+1.
